# Construction of the cervical cancer common terminology for promoting semantic interoperability and utilization of Chinese clinical data

**DOI:** 10.1186/s12911-021-01672-x

**Published:** 2021-11-16

**Authors:** Na Hong, Fengxiang Chang, Zhengjie Ou, Yishang Wang, Yating Yang, Qiang Guo, Jianhui Ma, Dan Zhao

**Affiliations:** 1 National Cancer Center/National Clinical Research Center for Cancer/Cancer Hospital, Chinese Academy of Medical Sciences and Peking Union Medical College, Beijing, China; 2 Digital Health China Technologies Co., Ltd., Beijing, China

**Keywords:** Cervical cancer, Common terminology, Interoperability, Clinical terms, Electronic health records

## Abstract

**Background:**

We aimed to build a common terminology in the domain of cervical cancer, named Cervical Cancer Common Terminology (CCCT), that will facilitate clinical data exchange, ensure quality of data and support large scale data analysis.

**Methods:**

The standard concepts and relations of CCCT were collected from ICD-10-CM Chinese Version, ICD-9-PC Chinese Version, officially issued commonly used Chinese clinical terms, Chinese guidelines for diagnosis and treatment of cervical cancer and Chinese medical book Lin Qiaozhi Gynecologic Oncology. 2062 cervical cancer electronic medical records (EMRs) from 16 hospitals, belong to different regions and hospital tiers, were collected for terminology enrichment and building common terms and relations. Concepts hierarchies, terms and relationships were built using Protégé. The performance of natural language processing results was evaluated by average precision, recall, and F1-score. The usability of CCCT were evaluated by terminology coverage.

**Results:**

A total of 880 standard concepts, 1182 common terms, 16 relations and 6 attributes were defined in CCCT, which organized in 6 levels and 11 classes. Initial evaluation of the natural language processing results demonstrated average precision, recall, and F1-score percentages of 96%, 72.6%, and 88.5%. The average terminology coverage for three classes of terms, clinical manifestation, treatment, and pathology, were 87.22%, 92.63%, and 89.85%, respectively. Flexible Chinese expressions exist between regions, traditions, cultures, and language habits within the country, linguistic variations in different settings and diverse translation of introduced western language terms are the main reasons of uncovered terms.

**Conclusions:**

Our study demonstrated the initial results of CCCT construction. This study is an ongoing work, with the update of medical knowledge, more standard clinical concepts will be added in, and with more EMRs to be collected and analyzed, the term coverage will be continuing improved. In the future, CCCT will effectively support clinical data analysis in large scale.

## Background

A terminology is a standard concept system that represents a set of concepts within a domain and the relationships among these concepts. Terminologies in the healthcare domain facilitate not only concept representation but also integration of healthcare knowledge and data. The diversity of clinical terms expression deters efficient sharing and use of medical knowledge and experience among hospitals and research institutions. In addition, it causes lack of semantic interoperability among health care information systems, eventually affecting implementation of clinical practices and secondary use of electronic health records (EHRs) data. To overcome these challenges, there are several health care terminologies have been adopted in clinical systems. They are usually developed to standardize specific types of healthcare data, such as disease, diagnosis, drug, procedure, laboratory test, nursing care, and insurance. Different standards have been developed to achieve distinct purposes and have a specific clinical or research emphasis. For example, disease ontology (DO) was developed to address data integration, standardization and annotation for human disease data [1]. The International Classification of Diseases, Tenth Revision (ICD-10) was developed mainly to encode diagnosis information [2]. Systematized Nomenclature of Medicine-Clinical Terms (SNOMED CT) is to encode general clinical observation data and it is widely used in many countries by licensing [3]. Gene Ontology (GO) provides structured, controlled vocabularies and classifications that use in the annotation of genes, gene products and sequences [4], and Logical Observation Identifiers Names and Codes (LOINC) [5], which originally developed to encode laboratory observations, has been expanded to also represent clinical observations.

Within the cancer domain, cancer researchers and clinicians have recognized the need for standardized terminology, and numerous studies and applications need terminologies to clarify communication among various types of knowledge and data. The development of cancer-related terminologies has become an important part of cancer studies and practices. Among various cancer-related ontologies or terminologies, the most widespread used systems are The National Cancer Institute’s Thesaurus (NCIt) [6, 7], which is a biomedical vocabulary that provides consistent, unambiguous codes and definitions for concepts and properties used in cancer research, clinical care, public health and administrative activities, and The International Classification of Diseases for Oncology, Third Edition (ICD-O-3) [8], which is a multiaxial classification used in cancer registries in order to record the topography and the morphology of a neoplasm. Many similar development and refinement work of cancer-related terminology has been implemented and evolved during the past decades [9].

Cervical cancer is ranked the fourth most common cancer among women worldwide and it is the second most common cancer among women in China, accounting for approximately 1,30,000 of all newly diagnosed cancers each year. In this specific domain, clinical terms and classifications used in clinical and research settings are highly inconsistent and ambiguous. For example, the terms used in clinical guidelines or existing terminology and ontologies have certain variations, possibly because they have been developed independently by different institutes. Moreover, over the years, the use of some terms has evolved and new terms have been introduced. The terminology challenges have become the main obstacles for a broad collaboration of cervical cancer researchers and clinical practitioners, and these challenges are continuously increasing with accumulation of medical data. Historically, there are some studies have been reported on cervical cancer related terminology standards, for example, the Bethesda System of cervical cytology was developed to provide a uniform system of terminology that would promote clear management guidelines [10], and the proposed cervical cytology terminology that allow European’s cervical cancer screening programs comparable with each other as well as with programs elsewhere in the world [11]. Although these previous studies provide experience for building cervical cancer related terminology, they only exhibit some aspects of cervical cancer terms with different structure and granularity. Although some introduction and translation work of international health care terminology standards have been conducted; however, introducing these international health care terminology standards to domestic clinical environment of China requires several localizations works and sometimes is not directly practicable. A paucity of efficient introduction and localization works of international vocabularies or terminology standards also exists. Thus, it is necessary to identify or to build a cervical cancer specific terminology that allows the integration of cervical cancer disease concept hierarchy, synonyms, semantic relations, etc., considering all kinds of existing terminology gaps. These challenges imply an immediate need to develop appropriate terminology standards specific to clinical environment of China.

On the other hand, China has various provincial level administrative regions and hospital tiers. Various of term expression habits and idioms exist in different regions and hospitals, which cause inconsistent term usage and bring huge challenges on utilizing clinical data across the sites. In order to meet domestic terminology needs, some Chinese medical terminologies have been developed, such as Chinese version of Medical Subject Headings [12], Chinese Traditional Medicine and Material Medical Subject Headings [13], etc. These terminologies are used to retrieve medical literature, represent medical knowledge, and indicate specific clinical domain data, and so on. However, they have not been widely utilized because of the limited term coverage and lacking efficient supporting information systems. Furthermore, to our knowledge, a comprehensive and practicable Chinese terminology for cervical cancer domain is currently in high demand in China.

Therefore, developing a common terminology of cervical cancer to promote large scale data analysis and data exchange between multiple sources is an essential work, especially for cervical cancer clinical research and practices. In this study, we aimed to develop and evaluate a domain terminology CCCT using various medical knowledge and EMRs from Chinese hospitals. The CCCT will effectively improve clinical data analysis capability in the use of EMRs, assure quality of data, and enhance interoperability between multiple data sources, regions and professionals.

## Methods

### Data sources

The acquisition of knowledge is key to establish a domain common terminology, which is the basis for constructing the CCCT. Sources of medical knowledge include medical records, books, diagnosis and treatment guidelines. In this study, the standard concepts, core terms, relations framework modeling works were manually curated by reviewing multiple sources knowledge, include Chinese medical professional books, existing terminologies and ontologies, such as ICD-10-CM Chinese Version, ICD-9-PC Chinese Version, officially issued commonly used Chinese clinical terms [14], Chinese guidelines for diagnosis and treatment of cervical cancer (National Health Commission of China released, 2018 version), Lin Qiaozhi Gynecologic Oncology 4th Edition and Pharmacopoeia of the People's Republic of China.

In addition, 2225 cervical cancer EMRs from 16 hospitals of China, belong to different regions and hospital tiers, were collected for terminology enrichment. Considering that the use of terms differed between regions, traditions, cultures, and language habits within China, clinical terms were collected by choosing EMRs from scattered location areas and hospital tiers.

### Methodology framework

**Fig. 1:**
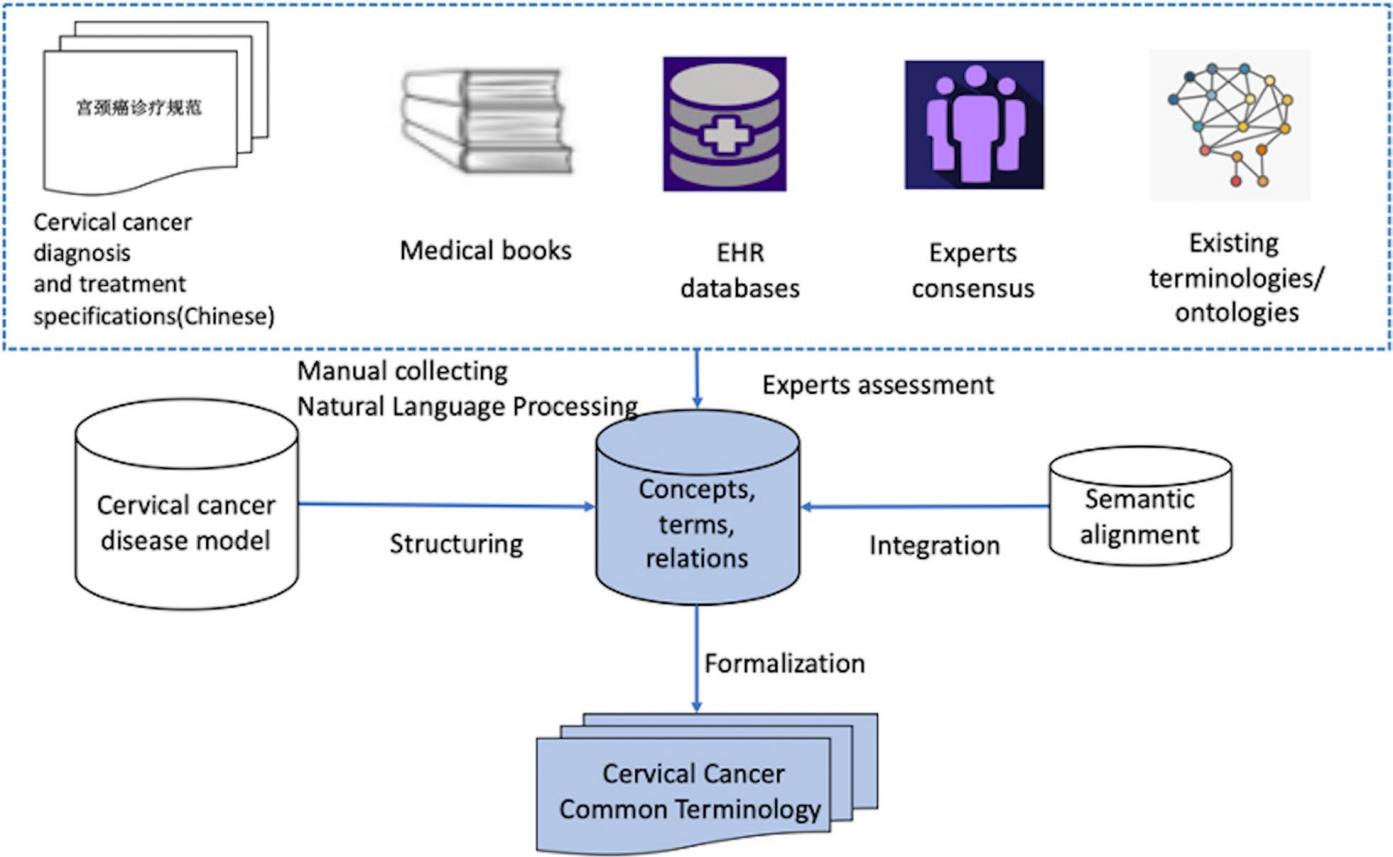
The methodology framework of building cervical cancer common terminology

To build the CCCT, we used multiple methods to acquire and model cervical cancer knowledge, including information extraction, semantic alignment, formalization, and consensus assessment. Furthermore, we developed a cervical cancer concept model that provided a schema for structuring, formalizing, and integral representation of these terms. The schema is shown in Fig. 1. The construction of CCCT in our study mainly includes three steps: concept modeling and clinical terms collections and semantic alignments. First, the cervical cancer standard concepts were extracted from the existing resources, such as clinical guidelines and medical professional books, and integrated into a terminology framework. Second, the clinical terms were extracted from clinical notes by using machine learning-based and rule-based natural language processing (NLP) methods. Third, the selected elements were integrated by semantic alignment to build a cervical cancer common terminology.

### Creating concept model from existing medical knowledge

Automated methods help process high volumes of text; however, they are not fully efficient and accurate in knowledge modeling. Existing domain knowledge and experts’ involvement are essential to building a concept model. The cervical cancer concept model was created mainly by manually reviewing of existing knowledge and assessing by clinical experts. First, we identified the key domain concepts, common terms, various expressions from existing guidelines and medical books. Next, we reused the already formalized knowledge by performing a search of the existing ontologies and terminologies, including ICD-10-CM Chinese version and ICD-9-PC Chinese Version, etc. Searching ontologies and terminologies helped reuse widely accepted existing, relevant, conceptualized and well-maintained knowledge. Then, an interview with clinical experts was performed after initial building. An iteratively improvement and review of these steps was conducted to generate a concept model of cervical cancer.

### Terminology enrichment from EMRs using natural language processing

**Fig. 2:**
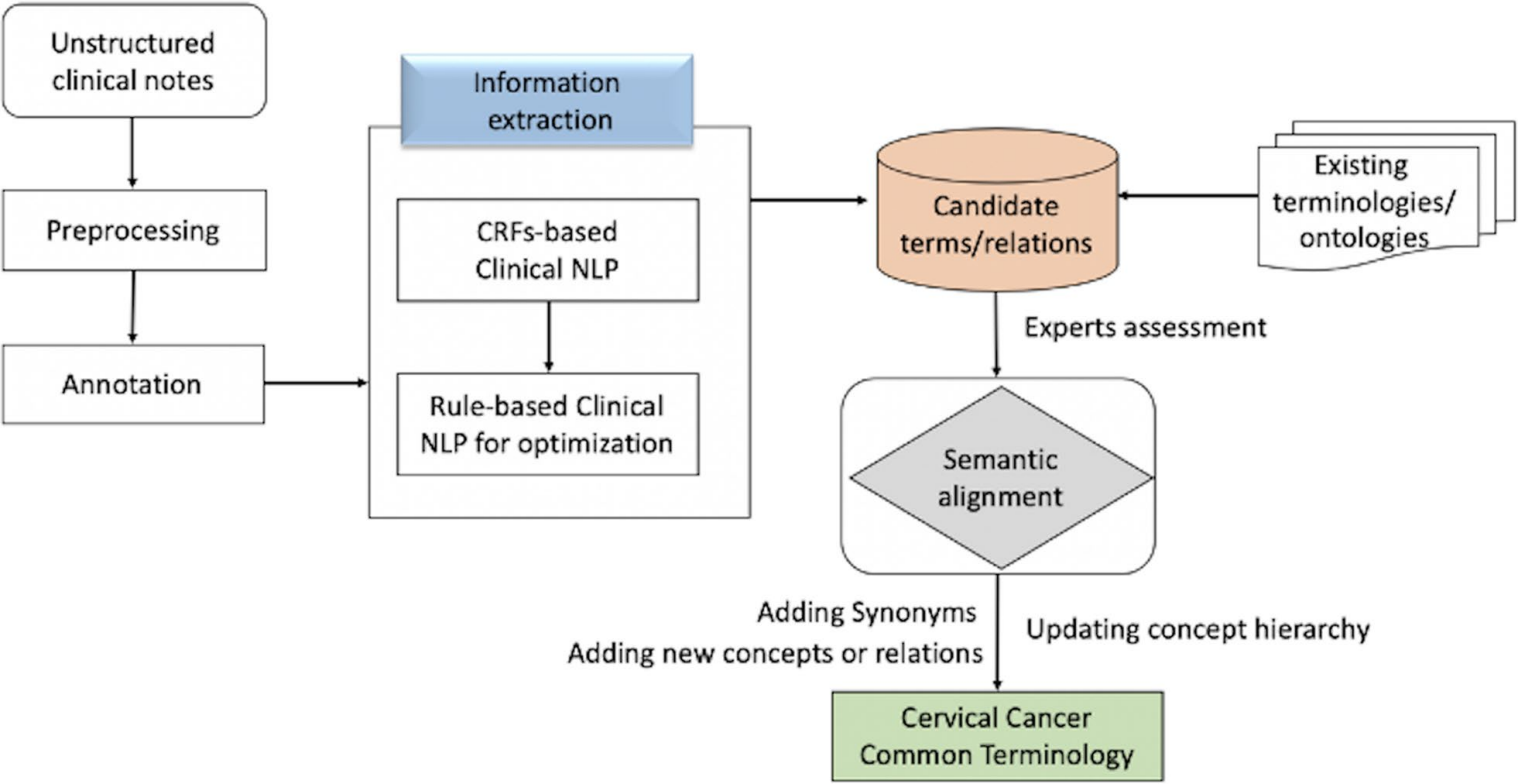
The clinical natural language processing supported information extraction for terminology enrichment

EMRs contain various clinical observations about patients such as patient states, diseases, treatments, and laboratory results. Unstructured clinical notes, with several clinical observations and results written by the clinicians, contain various clinical expressions. In our study, entities and relations were automatically extracted from clinical notes by using NLP technologies. A combination method of conditional random fields and rule-based information extraction was used to recognize named entities and relations from unstructured clinical notes. All extracted data were subjected to quality check and reviewed by medical experts. The flowchart is displayed as Fig. 2.

1. Information extraction based on conditional random fields

The conditional random field (CRF) model is an undirected statistical graph model. Essentially, it expresses a probability distribution that depends on a large number of random variables through the product of local functions that depend on a few variables. Because CRFs do not have strict independent assumption, the features can be selected arbitrarily. Therefore, the method of global normalization is adopted, avoiding the problem of label bias. In addition, as needed, researchers can add custom features, such as dictionary, part-of-speech, and word clustering, which makes it widely adopted. Currently, CRFs are successfully being applied to many clinical NLP studies, such as Named Entity Recognition (NER) and relationship extraction.

The CRF model can calculate the conditional probability of the specified output node when provided with the value of the specified input node. A specific NER task estimates the most likely label sequence based on the known sequence. If observation sequence is $O= <{o}_{1}, {o}_{2}, \dots {o}_{T}>$, then the conditional probability of the state sequence $S=<{s}_{1}, {s}_{2}, \dots {s}_{T}>$ is:

1 $${P}_{\Lambda }\left(s|o\right)=\frac{1}{{Z}_{0}}\mathrm{exp}\left(\sum_{t=1}^{T}\sum_{k}{\lambda }_{k}\times {f}_{k}\left({s}_{t-1}, {s}_{t}, o, t\right)\right)$$

where ${f}_{k}\left({s}_{t-1}, {s}_{t}, o, t\right)$ is the feature function and ${\lambda }_{k}$ is the parameter obtained by training model. The value range of the feature function is $-\infty , \cdots , +\infty$. To add all conditional probabilities up to 1, we added the following normalization factors:

2 $${Z}_{0}=\sum_{s}\mathrm{exp}\left(\sum_{t=1}^{T}\sum_{k}{\lambda }_{k}\times {f}_{k}\left({s}_{t-1}, {s}_{t}, o, t\right)\right)$$

Similar to the hidden Markov model, the value of Z_0_ can be obtained through dynamic programming. To train the CRF model, given the observation sequence, the maximum likelihood function L_Λ_ can be obtained by maximizing the state sequence:

3 $${L}_{\Lambda }=\sum_{i=1}^{N}\mathrm{log}\left({P}_{\Lambda }\left({s}^{\left(i\right)}|{o}^{\left(i\right)}\right)\right)-\sum_{k}\frac{{\lambda }_{k}^{2}}{2{\sigma }^{2}}$$

$where \left\{\langle {o}^{\left(i\right)}, {s}^{\left(i\right)}\rangle \right\}$ represents the labeled training data [15].

**Fig. 3:**
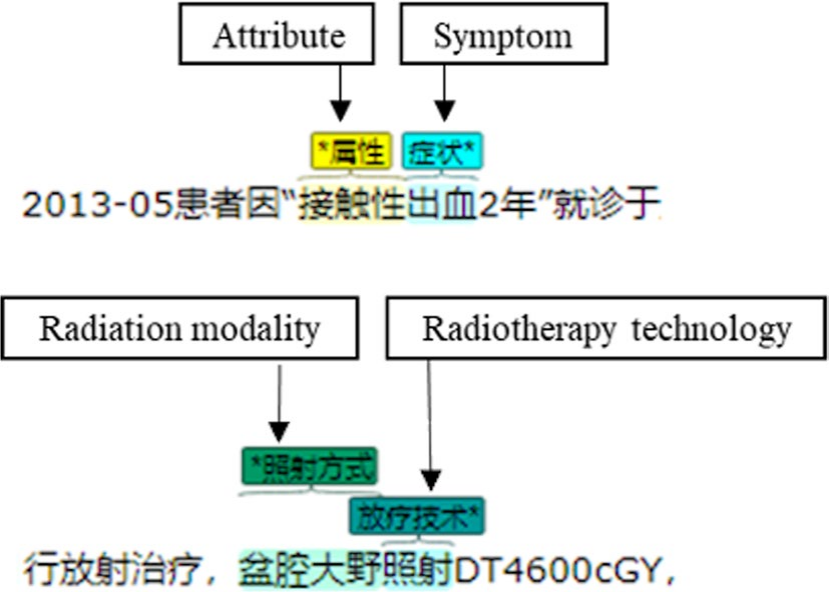
Examples of entity and relation annotations for natural language processing

The clinical notes were annotated by medical experts for NLP training and testing purpose. In addition to the regular NLP entity and relation annotations using above CRF model, we found that Chinese entities were often composed of two or more parts, called compound entity, which results in the uncertainty of the entity window length and likely affecting the accuracy of NLP results. For example, a compound entity with ‘attribute’ followed by ‘symptom’, and a compound entity with ‘radiation modality’ followed by ‘radiologic technology’, is shown in Fig. 3. To achieve annotation consistency, guidelines with inner agreements by our multidisciplinary team, includes clinical experts, knowledge engineers, and NLP engineers, were created to provide instructions to medical experts’ annotation work.

2. Refine NLP results based on rule-based methods

Since most of these compound entities have regular language patterns, we defined rules for each of these patterns. These rules then use regular expressions to identify specific instances of various common compound entities. Seven rules and common examples of compound entities are presented in Table 1. We iteratively improved these rules with new compound entities and patterns discovered.

**Table 1:** Examples of regular expression (rules)

Regular expression	Example
Body part + symptom	阴道出血/Vaginal bleeding
Attribute + symptom	接触性出血/Contact bleeding
Body part + Attribute + symptom	阴道不规则出血/Vagina irregular bleeding
Attribute + body part + symptom	接触性阴道出血/Contact vaginal bleeding
Attribute + body part + Attribute + symptom	同房后阴道出血/Postcoital vagina irregular bleeding
Radiotherapy site + Radiotherapy technology	盆腔适形放射治疗Pelvic conformal radiotherapy
Radiation modality + Radiotherapy technology	骨盆大野放射治疗/Large field radiotherapy of pelvis

### Semantic alignment for conceptualization

Various expressions, original terms, existing reused concepts, and classification relationships obtained from multiple sources were reviewed and aligned by clinical experts with multi-round iterations until consensus was achieved. Concepts, terms and relations that contain the same semantics were aligned and formalized. For example, concept “血尿/ Urine with blood” has many different Chinese expressions “血尿/尿血/小便带血/小便时带血”, we manually reviewed these synonyms and aligned them into one concept. A specialized tool, Protégé, was used for terminology management.

### Evaluation methods

For systematically evaluation of NLP results, the annotated testing dataset was used as the reference standard. Precision, recall, and F1-score were used to evaluate the named entity recognition performance.

$${\text{Precision}} = \frac{{\text{Number of correctly recognized named entities}}}{{\text{Total number of recognized named entities by NLP}}}$$

$${\text{Recall}} = \frac{{\text{Number of correctly recognized named entities}}}{{\text{Total number of recognized named entities by clincial expert}}}$$

$${\text{F - measure}} = \frac{{2*{\text{Precision}}*{\text{Recall}}}}{{{\text{Precision}} + {\text{Recall}}}}$$

Furthermore, we evaluated terminology coverage to measure the terminology usability in the cervical cancer domain. The evaluation equation is as follows:

$${\text{Terminology coverage}} = \frac{{\text{Number of clinical terms matched with the terms defined in CCCT}}}{{\text{Number of all the clinical terms in the testing corpus}}}$$

## Results

### Term variety analysis

By manually analyzing multiple sources of terms, we found that there were variety in Chinese cervical cancer terms, and these terms usage in clinical settings was highly common. These common terms need to be collected and mapped to standard concepts. Some term diversity examples are listed below.

1. Standard concepts are sometimes replaced by common terms or abbreviations to use in real communication. For example, “宫颈锥切/cervical cone cutting” actually means “子宫颈锥形切除术/conization of cervix” and “盆腔MRI/ pelvic MRI” is an abbreviation of “盆腔磁共振成像/ pelvic magnetic resonance imaging”.
2. Patient manifestation and disease status may have different expressions. For example: the recurrence and metastasis status of “侵犯阴道壁/ Invasion of the vaginal wall” in clinical settings are written in different expressions, such as “累及阴道壁/ Involving the vaginal wall”, “阴道受累/ Vagina involvement”, or “阴道受侵/ Vaginal invaded”.
3. The common name and the trade name of a drug are sometimes mixed. For example, “奥沙利铂 + 卡培他滨/oxaliplatin + capecitabine” is written as “艾恒 + 卡培他滨/L-OHP + capecitabine” and “多西他赛 + 卡铂/docetaxel + carboplatin” is written as “多西他赛 + 伯尔定/docetaxel + BED” or “艾素 + 卡铂/taxotere + carboplatin”.

### Hierarchy and content of CCCT

**Fig. 4:**
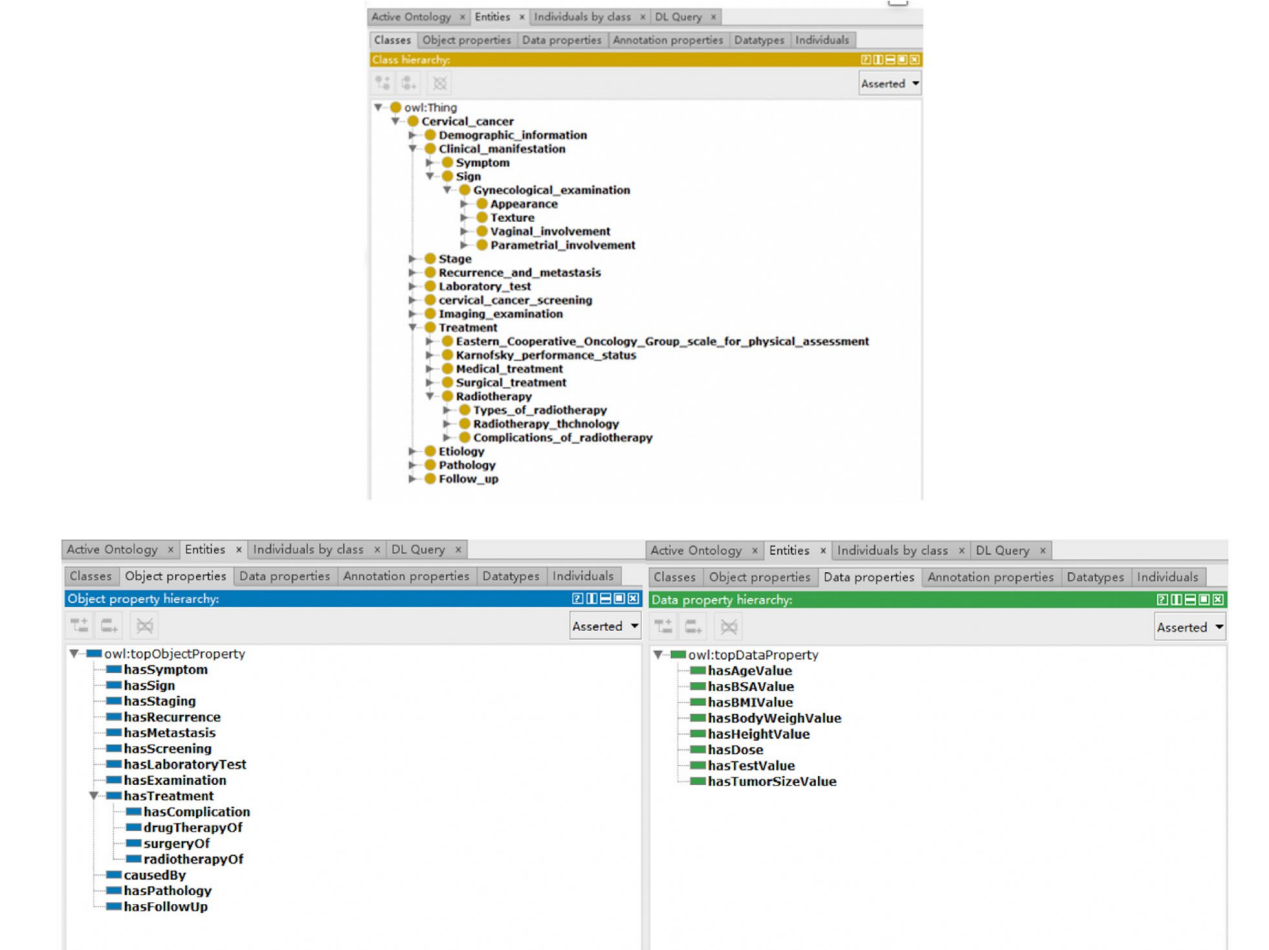
The hierarchy of cervical cancer common terminology

The common terminology was organized into a hierarchical structure of tree relationships, with a total of six levels under the guide of clinical experts. Among them, the 11 categories were clinical manifestation, demographic information, stage, recurrence and metastasis, laboratory test, cervical cancer screening, imaging examination, treatment, etiology, pathology, and follow-up. These 11 categories branched downward in a parent–child relationship. Additionally, 16 relations and 6 attributes were defined, such as Has_staging, Drug_therapy_of, etc. Concepts hierarchies and relationships were built using Protégé. As presented in Fig. 4.

The terminology is curated by a combination method of NLP technology and experts’ review. After various terms obtained from multiple sources and aligned by our multidisciplinary team, the CCCT was created with 2062 terms in total and include clinical manifestation (284), demographic information (14), stage (44), recurrence and metastasis (81), laboratory test (43), cervical cancer screening (76), imaging examination (49), treatment (1316), etiology (38), pathology (182) and follow-up (28), as list in Table 2.

**Table 2:** Distributions of the common terminology of cervical cancer

Concept	Number of standard concepts	Number of common terms	Total
Clinical manifestation	45	244	289
Demographic information	14	3	17
Stage	43	0	43
Recurrence and metastasis	32	41	73
Laboratory test	43	0	43
Cervical cancer screening	20	70	90
Imaging examination	15	4	19
Treatment	575	691	1266
Etiology	34	2	36
Pathology	36	125	161
Follow-up	28	0	28
Total	880	1182	2062

### Evaluation results

The technical methods and usability of CCCT was evaluated by the following two parts.

1. NLP results evaluation. Surgery reports were selected for evaluating the NLP results, and the results of the nine typical entities or modifier types, namely, lymphatic or blood vessel invasion, symptoms, radiation mode, attributes, radiotherapy technology, body parts, surgical procedure, body parts of radiotherapy, and negation, were selected for evaluation. The training and testing corpus were manually annotated and cross examined by two clinical experts. We randomly selected four specific entity types (cervical bleeding, surgery name, radiotherapy technology, and vascular tumor thrombus) for performance evaluation, the results are presented in Table 3.
2. Terminology coverage evaluation. To evaluate the terminology coverage in different hospital regions and tiers, 445 clinical notes that were randomly selected from 16 hospitals to evaluate the terminology coverage. The terminology coverage evaluation results on three types of terms: clinical manifestation (87.22%), treatment (92.63%), and pathologies (89.85%).

**Table 3:** Evaluation results of natural language processing

Entity type	Number of recognized entities	Number of recognized entities by NLP	Number of recognized entities by experts	Precision	Recall	F1-score
Clinical manifestation: 阴道出血/cervical bleeding	116	122	126	0.951	0.921	0.936
Treatment: 手术名称/surgery name	171	178	176	0.961	0.972	0.966
Treatment: 放射治疗技术/radiotherapy technology	135	177	164	0.763	0.823	0.791
Pathology: 血管瘤栓/vascular tumor thrombus	68	74	70	0.917	0.971	0.944

## Discussion

This terminology study effort was a novel attempt in Chinese cervical cancer domain, and the results demonstrated the feasibility of building an applicable domain common terminology by utilizing the proposed methods. The CCCT meets the need of cervical cancer research and practices in China, and revealed the real Chinese medical terms status. The advantage of our study is machine learning enabled NLP technologies were used to deal with large amounts of EMR data, which collected from multiple hospital nationally and ensure the terminology coverage in real world data; furthermore, the construction methodology and implementation pipeline could be applied to other medical domains and specialties.

There are still many challenges to overcome in clinical terminology studies. The clinical term expressions vary between different parties because of linguistic variations in different settings, such as among researchers, physicians, pathologist, surgeons, radiologists, anatomists, and others; furthermore, expression differences exist between regions, traditions, cultures, and language habits within the country, and these variations lead to interoperability challenges among health care systems, providers, and vendors. This requires more medical knowledge and clinical data to be collected for analysis in next step. For example, compared with other entity types, the ‘radiotherapy technology’ is with relatively low performance for the reason that the rapid development of new radiotherapy technologies make it is easy to be expressed diversely and inaccurately in clinical usage. Furthermore, the treatment and regimen of cervical cancer in China is complex, and the comprehensive treatment of early, middle and late stages, as well as the comprehensive treatment of surgery, radiotherapy and chemotherapy, leads to relatively complex regimens. In addition, directly introducing international terminology standard is also impracticable for Chinese cervical cancer term environment, because the introduced international terminology may not fully cover Chinese clinical terms. Using clinical manifestations related terms as examples, our initial results showed that only 35 from 72 (48.6%) frequently used terms could be mapped to SNOMED CT. All these statuses demonstrated the high demands and challenges of developing a Chinese clinical terminology of cervical cancer.

For the terminology coverage, evaluation result of “treatment” was 92.63%, indicating that most of the treatment related names mentioned in EMRs were covered by the common terminology. However, the coverage of “clinical manifestation” was 87.22% and “pathologies” was 89.85%, indicating that there are some peculiar term usages and habits in these domains, and the common terminology still did not cover a small part of the term expressions. However, compared with the evaluation results that only using standard concepts, the extended common terms highly improved the terminology coverage. The improved percentage are: “clinical manifestation” was from 44.73 to 87.22%, “treatment” was from 40.37 to 92.63% and “pathologies” was from 41.75 to 89.85%, which will highly improve future clinical data utilization.

There are limitations in this study. The hospital EMR data that collected in this study was still not enough; Besides, EMR data annotation is quite labor-intensive, with the purpose of implementing effective validation and evaluation of our methods, the extracted and evaluated NLP results mainly focused on four entity types within clinical manifestation, treatment and pathology. In the future, more terms and relations will be annotated, extracted and evaluated, and more EMR data collected from multiple regions will be included to improve the terminology coverage and its usability in China. The terminology coverage will be continuously improved.

Compared with the development of international medical terminology and standards, China needs more efforts on studies and implementations of terminologies and standards. It is of critical importance to address the terminology gaps among huge amount of medical knowledge and clinical data in China. Development and implementation of a common domain terminology is an effective method to overcome terminology gaps. Our long-term goal is to enroll all the Chinese common terms, semantic relations and various expressions among cervical cancer research and practices. Maintenance work are ongoing to update the terminology.

## Conclusions

This study highlighted the need for building a fundamental domain terminology for cervical cancer, especially under Chinese clinical environment, and conducted a terminology implementation and evaluation to better support clinical data analysis, semantic interoperability and data quality in the domain of cervical cancer.

## Data Availability

Please contact zhaodan@cicams.ac.cn for CCCN sharing and research.

## Abbreviations

CCCT: Cervical Cancer Common Terminology
EMRs: Electronic Medical Records
EHRs: Electronic Health Records
DO: Disease Ontology
ICD-10: The International Classification of Diseases, Tenth Revision
SNOMED CT: Systematized Nomenclature of Medicine-Clinical Terms
GO: Gene Ontology
LOINC: Logical Observation Identifiers Names and Codes
NCIt: The National Cancer Institute’s Thesaurus
ICD-O-3: The International Classification of Diseases for Oncology, Third Edition
NLP: Natural Language Processing
CRF: Conditional Random Field
NER: Named Entity Recognition
